# Approaching microaggressions toward ​a career in pathology: A potential path to a respectful culture

**DOI:** 10.1016/j.acpath.2026.100295

**Published:** 2026-09-11

**Authors:** Lydia Pleotis Howell

**Affiliations:** a Department of Pathology, Laboratory Medicine, University of California, Davis School of Medicine, Sacramento, CA, USA; b Association for Academic Pathology, Wilmington, DE, USA

**Keywords:** Microaggressions, Pathology careers, Professionalism, Workplace culture

Pathology has sadly had a long and unfair history of being perceived as a specialty for those without a good bedside manner, who cannot “cut it” in clinical medicine, or who want “easy” work hours because they are lazy or undedicated to patient care. In their recent article, George et al. have documented these negative perceptions and several others through their survey which provides unique insights from students regarding attitudes of other specialties toward pathology.^1^ ​Notably, they point out the challenges medical students face regarding their educational experiences and journey toward our specialty when they share their specialty interest.

The negative and unprofessional remarks reported by students in this article are likely familiar to all of us in pathology. What may not be familiar, however, is recognition of these comments as microaggressions, which are most commonly associated with “put-downs” toward racial and ethnic minorities. Microaggressions have been defined as the brief, sometimes subtle, everyday exchanges that either consciously or unconsciously disparage others based on personal characteristics or perceived group membership and usually involve marginalized groups.^2^ ​Though pathologists are not a marginalized group in the same sense as racial and ethnic groups who are under-represented in medicine, the comments shared in this article nonetheless demonstrate assumptions of inferiority and second-class citizenship of pathologists as physicians. These comments can lead to marginalization of pathologists as members of the healthcare team and can create stress in both educational and work settings, adversely impacting performance, professional identity, and choice of pathology as a career due to messages that the pathologist’s role and expertise is not valued.

How can those of us in pathology—especially pathology faculty members—help address microaggressions toward our specialty and create a more supportive, professional, and respectful culture that will benefit our students and attract more talent to our field? My colleagues at University of California, Davis, Kupiri Ackerman-Barger, and Negar Nicole Jacobs, have created the Microaggressions Triangle Model^3^ which has been widely adopted in academic health care settings. In this model, responses to microaggressions are approached from three distinct perspectives: the recipient, the bystander, and the source. Since pathology faculty often serve as advisors and mentors to students interested in pathology careers (i.e., the recipients of microaggressions), we must be prepared to hear and even draw out these concerning experiences and coach students on appropriate responses to the “digs” that they may receive. Students can navigate the power differential of faculty in other specialties who make disparaging comments by demonstrating curiosity and asking neutral but clarifying questions such as “I want to better understand what you meant by that comment—can you explain it to me?” Appealing to common values emphasized on clinical rotations can also be effective: “I know you care about the healthcare team in the delivery of care; aren’t pathologists part of this team?” As faculty, we may also be bystanders to negative comments to students or to our pathology colleagues which means we must be prepared to speak up as well. Sometimes, it is hard to respond in the moment—no one wants to pick a fight publicly—and it is also not uncommon to question oneself regarding what was actually said. Nonetheless, providing feedback to the offender is important whether immediate or shortly after thinking through the experience. Discussing the incident with a trusted friend or colleague can also be helpful for both students and faculty when considering a response. The third point of the Microaggressions Triangle is the source. We may all be the source of microaggressions since these are often derived from unconscious biases. It is therefore important to have humility and be ready to recognize and apologize for any hurtful remarks and strive to be better in the future. This preserves important relationships and sets a good example to others.

Lastly, I was impressed by the substantial percentage of positive responses in the survey in the George et al.,^1^ article, especially from surgeons and surgical specialties—this is an opportunity to be leveraged. Departments might want to borrow an idea from the Association for Academic Pathology and give an annual or biannual Friends of Pathology Award to individuals or other departments or units that are active supporters of pathology activities or pathology as a profession. An award draws attention to important values such as professionalism, brings visibility as well as honor to those who positively reinforce these values, and inspires others to do the same. We all have an obligation to create a supportive and respectful work and learning environment, and grass-root ​approaches on the individual and departmental levels can make a difference.
